# Need for weight management in Switzerland: findings from National Blood Pressure Week 2009

**DOI:** 10.1186/1471-2458-11-473

**Published:** 2011-06-15

**Authors:** Thomas Volken, René Schaffert, Peter Rüesch

**Affiliations:** 1 Department of Health Professions, Zurich University of Applied Sciences, Winterthur, Switzerland

## Abstract

**Background:**

The Swiss Health Survey (SHS) provides the only source of data for monitoring overweight and obesity in the general population in Switzerland. However, this survey reports body mass index (BMI) based on self-reported height and weight, and is therefore subject to measurement errors. Moreover, it is not possible to differentiate between overall and abdominal overweight. In this study, we aimed to gain a better understanding of the need for weight management in the general population of Switzerland by exploring and comparing prevalence rates of BMI and waist circumference (WC) based on physical measurements by trained observers, based on data from the 2009 National Blood Pressure Week (NBPW).

**Methods:**

Sample selection was based on a one-stage cluster design. A total of 385 pharmacies representing 3,600 subjects were randomly selected from pharmacies participating in NBPW. BMI measures based on physical weight and height (NBPW) were compared with self-reported BMI measures from the SHS. BMI and WC measurements from NBPW were then used to produce population estimates of overweight and obesity.

**Results:**

BMI-based overall prevalence of overweight and obesity was 43.6%, which was 4.7% higher than the value based on the respective SHS data. Overweight and obesity were more common in men (54.3%) than in women (33.5%). However, the overall prevalence of increased WC in the general population was estimated to be 64.4%, with more women (68.4%) than men (60.1%) exhibiting a WC above the threshold. The prevalence of subjects requiring weight management in the Swiss population remained high, even after adjusting WC for false positive and negative cases.

**Conclusions:**

Firstly, it may be more appropriate for health promotion programs to address the wider group identified by WC, which includes subjects who need to reduce their weight, or gain no further weight. Secondly, the gender differences are reversed depending on the use of WC or BMI to identify subjects suitable for health promotion programs; more women than men are identified by WC, and more men than women using BMI. These differences should be accounted for in gender-specific health promotion programs.

## Background

The Swiss Health Survey (SHS) currently provides the only source of data for monitoring overweight and obesity in the general population in Switzerland. The SHS was first conducted in 1992 and is repeated every five years. The 2007 sample included over 19,000 subjects, aged 15 years or older. Subjects were randomly selected within private households and computer-aided telephone interviews were conducted [1]. However, two potential problems exist. First, data on body mass index (BMI) is derived from self-reported weight and height; respondents tend to overestimate their height and underestimate their weight, leading to under-reporting of BMI values [2-8], with consequent underestimation of the prevalence of overweight and obesity. Second, the SHS only uses BMI as a measure of overweight and obesity; because abdominal fat mass can vary substantially within a narrow range of total body fat or BMI, other methods in addition to the measurement of BMI would help to identify individuals at risk from overweight-related illnesses [9,10]. Empirical evidence suggests that waist circumference (WC) may be a better predictor of overweight-related illnesses than BMI [11-14], or should be used together with BMI [10,15,16].

Measures used in public health programs should be easy to administer and the results should be able to be translated into simple messages for the public. WC is both easy to administer and to explain.

Few studies have developed correction factors that can be applied to self-reported data to reduce the biases in BMIs [2,17,18], and the results appear to be discouraging [7]. While such correction factors have been proposed for the BMI values reported in the SHS [2], there are currently no studies estimating the prevalence of subjects in the Swiss population in need of weight management based on WC. Neglecting the central fat distribution indicated by WC may lead to serious misclassification issues when identifying subjects who need to control their weight, because a considerable number of subjects with a low BMI may still have a WC and waist:hip ratio above the threshold [19]. Consequently, the Swiss prevalence figures for subjects in need of weight management and at risk of overweight-related illnesses may be biased.

The current study used BMI and WC data from National Blood Pressure Week (NBPW) 2009 to reduce both measurement and classification errors, and to provide an estimate of the prevalence of subjects in need of weight management in the Swiss population.

## Methods

### Subject recruitment

This study was based on the cross-sectional, descriptive data from the 2009 NBPW. NBPW formed part of a prevention initiative by the Swiss Heart Foundation. Between June 2^nd ^and June 10^th ^2009, 800 pharmacies in the association of Swiss pharmacies, pharmaSuisse, offered a blood pressure check free of charge. Subjects 14 years and older were recruited on a walk-in basis (self-selection). The check included standardized measurements of blood pressure, BMI, and WC, and survey questions on medication, diet, physical activity and smoking. The NBPW and SHS data used for this study are openly available for scientific use and can be obtained through the regular distribution channels of the Federal Bureau of Statistics and the Federal Office of Public Health.

### Anthropometric measurements

All potential measurers received rudimentary training prior to performing the field study. This training consisted of a self-study manual providing detailed instructions on measurement procedures. Upon completion of the training, potential measurers had to pass a formal multiple-choice test to be accepted for the field work.

For the measurement procedures, all participating subjects were asked to remove their shoes and heavy outer garments (jackets, coats, jerseys, etc.). Their waist was measured using a non-stretchable tape over the lightly dressed abdomen, midway between the lowest rib and the iliac crest. Body height was measured using a stadiometer or measuring rod. Body weight was measured using calibrated scales. The large number of participating pharmacies meant that it was impossible to use the same equipment at every measurement site. Subjects were identified as overweight/obese (BMI ≥ 25.0 kg/m^2^) according to World Health Organization references [9]. WC ≥ 94.0 cm in men and ≥ 80.0 cm in women were taken as markers of central obesity [9,20]. Subjects were defined as being in need of weight management if they exceeded their respective WC thresholds, and were identified as needing to reduce weight, or gain no further weight [19].

### Study population and sampling

No sampling frame for subjects participating in the NBPW was available, and economic constraints limited the number of questionnaires that could be collected from the 800 pharmacies for subsequent data entry and analyses. One-stage cluster sampling was therefore used. A total of 385 pharmacies with a total of 3,600 subjects was randomly selected from the participating pharmacies. For the purpose of this study, the initial sample was further narrowed down to include only subjects 18 years old or older, and for whom a full set of anthropometric data was available. This left 380 pharmacies and 3,170 subjects. Comparisons with national census and cross-sectional SHS 2007 data (Table 1) suggest that subjects participating in the NBPW are more likely to be female (68%, p ≤ 0.001) and that both participating males (59.5 years, p ≤ 0.001) and females (57.4 years, p ≤ 0.001) tend to be older than the target Swiss population. The gender difference may reflect the traditional gender-specific division of labour (with women being more likely to do the daily shopping), and the fact that subjects participating in the NBPW are older (female: 6.1 years, male: 9.9 years) may reflect the increased probability of developing disease with increasing age. However, this does not necessarily mean that sicker people are more likely to go to a pharmacy in any given week, because shopping can easily be done by proxy. Nevertheless, the locations of the data collection points and the self-selection of participating subjects in the pharmacies may represent potential sources of bias.

**Table 1:** Average age, BMI and WC by gender in the SHS and NBPW samples*

	SHS		NBPW	
	**male**	**female**	**male**	**female**
variable	n = 8,017 44.8%	n = 9,862 55.2%	n = 1,015 32%	n = 2,155 68%

**age (years)**	49.6	51.3	59.5	57.4
	(17.2)	(17.9)	(15.1)	(17.0)
**BMI (kg/m2)**	25.4	23.7	26.2	24.5
	(3.6)	(4.3)	(3.7)	(4.3)
**WC (cm)**	-	-	98.8	88.0
			(11.3)	(12.4)

*Age, BMI and WC were based on unweighted arithmetic means. Source: National Blood Pressure Week 2009, pharmaSuisse.

### Design effects

The design effects [21] resulting from one-stage cluster sampling (Table 2) amounted to an average of 1.8 (BMI), 1.9 (WC) and 2.0 (WC≥94(80)). In general, the average design effects for men were almost three times as large as those for women, because the sample size for men was much smaller (n = 1,015) than that for women (n = 2,155). Furthermore, the sample sizes for the 18-29 (n = 34) and 30-39 (n = 64) age groups were particularly small.

**Table 2:** Design effects for BMI, WC and WC above threshold NBPW 2009*

type of estimate	average DEFF	max DEFF	min DEFF	average DEFT
**BMI**				
male & female	1.8	9.3	0.4	1.2
female	1.0	2.0	0.4	1.0
male	2.7	9.3	0.6	1.5
**WC**				
male & female	1.9	9.8	0.5	1.2
female	1.1	2.1	0.5	1.0
male	2.7	9.8	0.6	1.5
**WC > = 94 (80)**				
male & female	2.0	9.4	0.5	1.3
female	1.1	1.8	0.5	1.0
male	2.9	9.4	0.8	1.5

*Average DEFF (design effect) and DEFT (square root of design effect) values are grand means based on the arithmetic means of the age and gender groups. Source: National Blood Pressure Week 2009, pharmaSuisse.

### Weighting, classification and adjustment of data

Data were standardized, i.e., weighted by post-stratification adjustment factors [22] including gender and age, to represent the Swiss population in 2007, with the exception of the sparsely populated cantons Uri and Appenzell Innerrhoden, which did not participate in the NBPW. The total population represented by the NBPW sample amounts to 6,103,469 inhabitants, compared to 6,143,378 inhabitants in all cantons.

**Figure 1:**
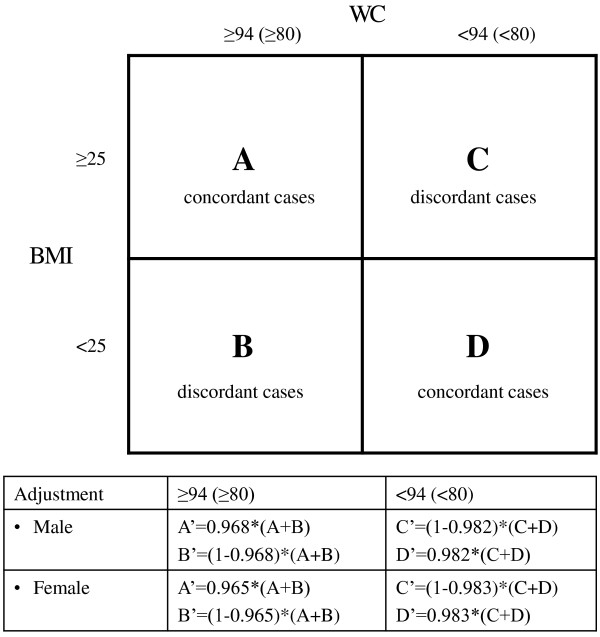
**Classification of Subjects in Need of Weight Management**.

Subjects were classified as being in need of weight management if their WC exceeded the threshold value. The sensitivity and specificity figures reported by Lean, Han and Morrison [19] were used to account for false positive and false negative cases: female subjects with a WC above the threshold were weighted with an adjustment factor (sensitivity) of 0.965 (true positive) and 1-0.965 (false positive), while male subjects were weighted with 0.968 and 1-0.968, respectively. Subjects below the critical WC threshold were weighted accordingly using the above specificity figures (female: 0.983, male: 0.982). Following Lean and colleagues, the prevalence rates for subjects in need of weight management, after adjusting for misclassification, reflected the combined prevalence of concordant cases identified by BMI and WC above the thresholds, as well as discordant cases having a BMI below the threshold, but a waist:hip ratio ≥ 0.95 (men) or ≥ 0.80 (women) (Figure 1). Most subjects with a high WC and a BMI below the cut-off also have a high waist:hip ratio, thus justifying weight management, while subjects with a BMI above and a WC below the thresholds have low waist:hip ratios [19].

### Statistical analyses

Statistical analyses were based on frequency tables; differences between categories were assessed using the design-based F-test, which is an F approximation to the second-order Rao-Scott Chi-squared statistics [23-25]. Statistical analyses also included a calculation of crude prevalence rates for overweight and obesity and increased WC by age group and gender. The resulting BMI values were compared with BMI data based on the SHS to assess the plausibility of the NBPW data. Two sample Student's *t*-tests with variance corrected for the design effect were used to assess BMI differences between the two surveys. To incorporate information on the appropriate weights and sampling units for correct variance estimation, all statistical analyses of the NBPW data were carried out using Stata's command for complex surveys ("svy prefix"). Statistical significance was established at p < 0.05.

## Results

### Self-reported and measured BMI

The population-corrected average BMI values based on height and weight measured by trained observers in the pharmacies were consistently higher than the BMI values based on self-reported weight and height over all age groups and both genders (Tables 3 and 4). The arithmetic mean of measured BMI was 24.0 kg/m^2 ^for women and 25.8 kg/m^2 ^for men, while the self-reported values were 23.6 kg/m^2 ^and 25.3 kg/m^2^, respectively (p < 0.001 for both men and women). The trajectory of mean BMI displayed a similar pattern over all age categories using either data source. BMI in women tended to be higher in older age cohorts, and the mean BMI was lower than in the preceding group only in the oldest age category (≥ 80 years). The BMI pattern was similar in men; however, a fall in average BMI was already achieved in the 70-79-year-old group, and the fall was greater in the oldest age category.

**Table 3:** Average BMIs SHS 2007^1^

	age	n	N	BMI	95% CI
**female**	18-29	1,214	564,162	21.7*	21.5-21.9
	
	30-39	1,762	572,102	23.1	22.8-23.3
	
	40-49	1,820	630,493	23.3***	23.0-23.5
	
	50-59	1,572	500,548	24.3	24.0-24.5
	
	60-69	1,642	420,054	24.8*	24.5-25.0
	
	70-79	1,225	297,687	25.3	25.0-25.6
	
	> = 80	627	159,492	24.3*	23.9-24.7

**male**	18-29	1,079	576,393	23.5*	23.3-23.8
	
	30-39	1,455	548,201	25.0***	24.7-25.2
	
	40-49	1,687	627,945	25.7**	25.5-25.9
	
	50-59	1,311	501,934	26.1**	25.8-26.4
	
	60-69	1,277	405,718	26.4*	26.1-26.6
	
	70-79	849	240,490	26.0	25.6-26.4
	
	> = 80	359	98,160	25.4	25.0-25.8

**total**		17,879	6,143,378	24.4***	24.3-24.5

**female**		9,862	3,144,538	23.6***	23.4-23.7

**male**		8,017	2,998,840	25.3***	25.2-25.4

^1^n: number of subjects in the sample; N: estimated number of subjects in the Swiss population; BMI: body mass index kg/m^2^; CI: confidence intervals. All BMI figures are population corrected (weighted) and are based on arithmetic means within age and gender groups. SHS BMI was calculated using self-reported height and weight, asterisks denote significant group differences between average SHS BMIs and their corresponding NBPW BMIs (see Table 4).* p < 0.05; ** p < 0.01; *** p < 0.001. Source: Swiss Health Survey 2007, Federal Office of Public Health.

**Table 4:** Average BMIs NBPW 2009*

	age	n	N	BMI	95% CI
**female**	18-29	194	551,065	22.2	21.7-22.6
	
	30-39	146	547,343	23.2	22.6-23.8
	
	40-49	312	610,493	24.3	23.8-24.9
	
	50-59	420	493,190	24.4	23.9-24.8
	
	60-69	508	406,797	25.0	24.6-25.4
	
	70-79	394	301,560	25.4	24.9-25.9
	
	> = 80	181	232,027	24.7	24.1-25.2

**male**	18-29	34	558,698	23.8	22.5-25.2
	
	30-39	64	545,221	26.1	25.3-26.8
	
	40-49	160	621,985	26.1	25.6-26.7
	
	50-59	204	495,701	26.5	25.9-27.1
	
	60-69	272	384,989	26.6	26.1-27.0
	
	70-79	196	234,820	26.3	25.8-26.8
	
	> = 80	85	119,580	25.5	24.8-26.2

**total**		3,170	6,103,469	24.9	24.6-25.1

**female**		2,155	3,142,475	24.0	23.7-24.2

**male**		1,015	2,960,994	25.8	25.4-26.2

*n: number of subjects in the sample; N: estimated number of subjects in the Swiss population; BMI: body mass index kg/m^2^; CI: confidence intervals. All BMI figures are population corrected (weighted) and are based on arithmetic means within age and gender groups. NBPW BMI was calculated using physical measurements of height and weight. Source: National Blood Pressure Week 2009, pharmaSuisse.

Self-reported average BMI values based on the SHS were significantly lower than average BMI values based on NBPW data in nine of the 14 age and gender groups (Table 3).

### Overweight and obesity and increased WC

Tables 5 and 6 summarize the prevalence of overweight and obesity (BMI ≥ 25.0 kg/m^2^) and the prevalence of central obesity above the threshold (WC ≥ 94.0 cm in men and WC ≥ 80.0 cm in women). Consistent with the above findings, the estimated prevalence of overweight and obesity in Switzerland based on measured NBPW BMI values was higher than that based on SHS data. The overall prevalence of overweight and obesity was 43.6%, which was 4.7% higher (p < 0.001) than the figure based on the respective SHS data (men: +5.8%, p < 0.001; women: +3.7%, p < 0.001). Overweight and obesity was more common in men (54.3%) than in women (33.5%; p < 0.001). However, the overall prevalence of increased WC in the general population was estimated to be 64.4%. More women (68.4%) than men (60.1%; p < 0.001) exhibited a waist circumference above the threshold. Unlike the prevalence of overweight and obesity, which decreased in the oldest (female) or two oldest age categories (male), the prevalence of above-threshold WC increased almost uniformly with increasing age, and reached ≥ 80% in both genders.

**Table 5:** Prevalence of overweight and obesity SHS 2007*

		BMI ≥ 25		
	**age**	**N**	**%**	**95% CI**
	
**female**	18-29	70,394	12.5	10.4-15.0
	
	30-39	130,732	22.9	20.5-25.3
	
	40-49	162,384	25.8	23.2-28.5
	
	50-59	187,396	37.4	34.4-40.6
	
	60-69	177,066	42.2	39.1-45.2
	
	70-79	147,086	49.4	45.9-53.0
	
	> = 80	61,850	38.8	34.2-43.6

**male**	18-29	155,242	26.9	23.7-30.5
	
	30-39	236,274	43.1	40.0-46.2
	
	40-49	337,913	53.8	50.8-56.8
	
	50-59	284,703	56.7	53.2-60.1
	
	60-69	254,060	62.6	59.3-65.8
	
	70-79	132,376	55.0	51.0-59.1
	
	> = 80	54,414	55.4	49.3-61.4

**total**		2,391,889	38.9	38.0-39.9

**female**		936,908	29.8	28.7-31.0

**male**		1,454,981	48.5	47.1-49.9

* N: estimated number of subjects with BMI≥25; %: estimated prevalence of BMI≥25; CI: confidence intervals. All figures are population corrected (weighted). Source: Swiss Health Survey 2007, Federal Office of Public Health.

**Table 6:** Prevalence of overweight and obesity and increased WC NBPW 2009*

		BMI ≥ 25			WC ≥ 94 (80)		
	**age**	**N**	**%**	**95% CI**	**N**	**%**	**95% CI**
	
**female**	18-29	99,419	18.0	13.2-24.2	258,489	46.9	38.5-55.6
	
	30-39	146,208	26.7	20.1-34.5	326,156	59.6	51.5-67.2
	
	40-49	219,151	35.9	30.6-41.6	418,736	68.6	62.9-73.8
	
	50-59	170,268	34.5	29.7-39.7	360,498	73.1	68.5-77.2
	
	60-69	177,773	43.7	39.7-47.8	327,520	80.5	76.5-84.0
	
	70-79	146,953	48.7	43.5-54.0	255,637	84.8	80.9-88.0
	
	> = 80	92,298	39.8	33.0-47.0	203,825	87.9	82.3-91.8

**male**	18-29	197,188	35.3	20.0-54.3	197,188	35.3	20.0-54.3
	
	30-39	323,725	59.4	47.7-70.1	332,244	60.9	49.7-71.2
	
	40-49	334,317	53.8	46.3-61.1	361,529	58.1	50.5-65.4
	
	50-59	298,879	60.3	52.9-67.3	320,748	64.7	57.5-71.3
	
	60-69	256,188	66.5	60.6-72.0	287,326	74.6	69.1-79.5
	
	70-79	142,569	60.7	53.8-67.2	185,700	79.1	73.1-84.0
	
	> = 80	53,459	44.7	34.6-55.3	95,664	80.0	69.0-87.8

total		2,658,395	43.6	40.5-46.7	3,931,259	64.4	60.8-67.9

female		1,052,071	33.5	30.8-36.3	2,150,861	68.4	65.2-71.5

male		1,606,324	54.3	48.9-59.6	1,780,398	60.1	54.4-65.6

* N: estimated number of subjects with BMI≥25 and WC≥94(80) respectively; %: estimated prevalence of BMI≥25 and WC≥94(80) respectively; CI: confidence intervals; WC: waist circumference. All figures are population corrected (weighted). Sources: National Blood Pressure Week 2009, pharmaSuisse & Swiss Heart Foundation; Swiss Health Survey 2007, Federal Office of Public Health.

### Subjects in need of weight management

Almost three quarters of the cases exhibited a concordant pattern with regard to BMI and WC (Table 7), i.e., both their BMI and WC were either above (40.7%) or below the thresholds (32.8%). Almost half of the men (48.6%) and one third of the women (33.3%) exceeded both threshold values, with markedly more men than women falling into this category (p < 0.001). In contrast, there was no significant gender difference in cases with BMI and WC both below the threshold values (female 31.3%; male 34.3%; p > 0.3). Both discordant categories showed significant gender differences; substantially more women (35.2%) than men (11.5%) had BMIs below and WCs above the threshold values (p < 0.001), while more men (5.6%) than women (0.2%) had BMIs above and WCs below the threshold values (p < 0.001). Overall, subjects with discordant threshold values accounted for 26.5% of the total population. However, approximately 90% of these cases were accounted for by having BMIs below and WCs above threshold values (23.7%), and little more than 10% were accounted for by the converse situation (2.8%).

**Table 7:** Prevalence of subjects in BMI and WC categories below and above threshold for weight management*

	female		male		total	
	
	% (**N**)	95% CI	% (N)	95% CI	% (N)	95% CI
**BMI ≥ 25**	33.3	30.5-36.1	48.6	43.5-53.8	40.7	37.7-43.9
**WC ≥ 94 (80)**	(1,045,299)		(1,439,830)		(2,485,129)	

**BMI < 25**	35.2	32.6-37.8	11.5	9.3-14.1	23.7	21.7-25.9
**WC ≥ 94 (80)**	(1,105,563)		(340,568)		(1,446,130)	

**BMI < 25**	31.3	28.4-34.5	34.3	28.6-40.3	32.8	29.3-36.4
**WC < 94 (80)**	(984,841)		(1,014,102)		(1,998,944)	

**BMI ≥ 25**	0.2	0.1-0.6	5.6	4.2-7.6	2.8	2.1-3.8
**WC < 94 (80)**	(6,772)		(166,494)		(173,266)	

**total**	100		100		100	
	(3,142,475)		(2,960,994)		(6,103,469)	

* N: estimated number of subjects in BMI/WC category; %: estimated prevalence of subjects in BMI/WC category in the Swiss population; CI: confidence intervals; WC: waist circumference. All values are population corrected (weighted). All BMI and WC values are based on arithmetic means within groups; BMI and WC were calculated using physical measurements of height and weight and waist circumference. Sources: National Blood Pressure Week 2009, pharmaSuisse & Swiss Heart Foundation.

The adjusted prevalence rates for subjects in need of weight management are given in Table 8. Essentially, this reflects the combined weighted prevalence of concordant cases with BMI and WC both above threshold values, and of weighted discordant cases (for details, see Methods). The need for weight management in Switzerland is thus determined to be high, with 62.2% of the Swiss population requiring weight management, representing fewer men (58.2%) than women (66.1%) who need to reduce their weight, or at least gain no more weight (p < 0.001). The need for weight management is generally more prevalent in older age cohorts. However, the age-related increase of prevalence rates is more uniform in women, in contrast to a slight drop in the need for weight management in 40-49-year-old men, compared to the preceding age cohort.

**Table 8:** Adjusted prevalence of subjects in need of weight management NBPW 2009

	Age	N	%	95% CI
	
female	18-29	249,442	44.2	35.9-52.9
	
	30-39	314,741	56.9	48.8-64.8
	
	40-49	404,080	66.2	60.4-71.6
	
	50-59	347,881	70.9	66.1-75.3
	
	60-69	316,056	78.8	74.5-82.5
	
	70-79	246,690	83.3	79.2-86.8
	
	> = 80	196,691	86.6	80.6-91.0
**male**	18-29	190,878	33.5	18.8-52.3
	
	30-39	321,612	59.0	47.7-69.5
	
	40-49	349,960	56.2	48.5-63.5
	
	50-59	310,484	62.9	55.6-69.6
	
	60-69	278,132	73.1	67.4-78.1
	
	70-79	179,757	77.7	71.5-82.9
	
	> = 80	92,603	78.7	67.2-86.9

**total**		3,799,006	62.2	58.5-65.8

**female**		2,075,581	66.1	62.7-69.2

**male**		1,723,425	58.2	52.4-63.8

* N: estimated number of subjects in need for weight management [adjusted WC≥94(80)]; %: estimated prevalence of subjects in need for weight management in the Swiss population; CI: confidence intervals. All figures are population corrected (weighted). Sources: National Blood Pressure Week 2009, pharmaSuisse & Swiss Heart Foundation; Swiss Health Survey 2007, Federal Office of Public Health.

## Discussion

BMI values based on SHS and NBPW data are both potentially subject to biases; however, the sources of these biases may be quite different. On the one hand, the self-reported nature of the SHS BMI data almost certainly include measurement errors due to factors such as the lack of standardized measurement instruments and procedures, respondents' knowledge about and recollection of their actual height and weight, response acquiescence, and social desirability [4,26-29]. On the other hand, measured NBPW measures of BMI and WC may be biased due to the self-selection of participating pharmacies and/or subjects, or the varying brands and qualities of the measurement equipment used [30]. A further limiting factor of this study may be its specific sample design. The average design effect was quite large, which may represent a problem in the case of male age cohorts with small sample sizes. However, despite the different sources of measurement errors and biases, there were substantial similarities between self-reported and measured BMI, and our findings were generally consistent with previously reported studies, which lend plausibility to the newly collected NBPW data. Firstly, the average BMI trajectory showed a similar pattern over all age categories for males and females using both data sources. BMI is generally higher in older age cohorts, and decreases again only in the highest age categories [31]. Secondly, measured BMI was consistently higher than self-reported BMI, which is a well-documented phenomenon in the research literature [3,5-7,28,32], though the difference was only significant in nine out of the 14 age and gender groups. Thirdly, average differences between measured and self-reported BMI values were similar in women (0.34 kg/m^2^) and men (0.37 kg/m^2^), contrary to the thesis suggesting that gender-specific differences exist in terms of self-declaration of weight and height, i.e., the idea that women may be more prone to report weights or heights to conform to socially constructed body images [5,29,33]. Not surprisingly, the estimated prevalence of overweight and obesity (BMI ≥ 25.0 kg/m^2^) in the Swiss population was significantly higher when based on NBPW BMI values (43.6%), compared to SHS values (38.9%), and the magnitude of the increase (4.7%; p < 0.001) was comparable with that in previous studies that assessed self-reported versus clinical measurements of BMI [6]. The prevalence of increased WC was even higher (64.4%). However, we found that the prevalence of overweight and obesity defined by BMI was higher in men (54.3% versus 33.5%; p < 0.001), whereas the prevalence of an increased WC was higher in women (68.4% versus 60.1%; p < 0.001) [13,34,35]. These findings have important implications. Firstly, prevalence figures for the population at risk of overweight and obesity and overweight-related illnesses based on SHS BMI data potentially understate the problem. Secondly, these prevalence figures are gender-biased. Only 25.1% of men with BMI below the threshold had a WC above the threshold, while 52.8% of women with BMI < 25 had a WC above the threshold. At the same time, more men (48.6%) than women (33.3%; p < 0.001) showed concordant above-threshold values for both BMI and WC. Hence, health promotion programs targeting individuals with a BMI above the threshold would miss over a third of the female population at risk. Thirdly, WC as a marker of abdominal overweight and obesity (visceral adipose tissue) has been reported to be more predictive for increased overweight-related mortality and morbidity risks, and identifies persons at increased cardiovascular risk better than the BMI [11,13,14,36]. However, other studies have supported the use of BMI [37], or found no differences between the predictive powers of WC and BMI [16].

For the above reasons, we used WC to identify subjects in need of weight management, i.e., to identify subjects who should reduce their weight, or who should gain no further weight. The adjusted total prevalence of subjects in need of weight management in the Swiss population was estimated to be 62.2%, with more women (66.1%) than men (58.2%; p < 0.001) requiring weight management. The potential implications of these findings are manifold, i.e., the risk of overweight-related illnesses and the associated cost burden are much higher than previously estimated, and weight-related health promotion programs should be equally targeted at men and women.

## Limitations of the study

This study had several limitations. Firstly, the sample consisted of subjects who selected themselves to be part of the study. Although we attempted to correct for known systematic sources of self-selection bias (age and gender) by applying post-stratification weightings, other, other unknown sources of self-section bias may still have been present. Secondly, the one-stage cluster sample design meant that the average design effect was quite large, especially in the case of male age cohorts with only a small number of subjects in the sample. Thirdly, the use of different measurement equipment (scales, tapes, rods) may have resulted in a greater measurement error than is generally desirable for clinical research. Finally, subjects were lightly dressed, potentially leading to slight over-estimations of WC and weight.

## Conclusions

The findings of this study differ from those of previous epidemiological studies based on SHS data in several respects. This may have various implications for public health and health promotion programs. Firstly, the prevalence of subjects with above-threshold WC was higher than the prevalence of those with above-threshold BMI. These differences mean that different numbers of people would be estimated to require weight management, depending on the parameter used. For health promotion programs, it may be more appropriate to address the wider group identified by WC, which comprises subjects who should reduce their weight, or gain no further weight. Secondly, the gender differences are reversed when WC or BMI is used to identify subjects suitable for health promotion programs; the former identifies more eligible women than men, while the latter identifies more men than women. These differences should be taken into account in gender-specific health promotion programs.

## Competing interests

The authors declare that they have no competing interests.

## Authors' contributions

TV contributed most of the writing and the analysis. RS and PR contributed to the writing and editorial review. All authors read and approved the final manuscript.

## Pre-publication history

The pre-publication history for this paper can be accessed here:

http://www.biomedcentral.com/1471-2458/11/473/prepub
